# A 16S rRNA gene sequencing and analysis protocol for the Illumina MiniSeq platform

**DOI:** 10.1002/mbo3.611

**Published:** 2018-03-25

**Authors:** Monica Pichler, Ömer K. Coskun, Ana‐Sofia Ortega‐Arbulú, Nicola Conci, Gert Wörheide, Sergio Vargas, William D. Orsi

**Affiliations:** ^1^ Department of Earth and Environmental Sciences, Paleontology & Geobiology Ludwig‐Maximilians‐Universität München Munich Germany; ^2^ GeoBio‐Center^LMU^ Ludwig‐Maximilians‐Universität München Munich Germany; ^3^ SNSB ‐ Bayerische Staatssammlung für Paläontologie und Geologie Munich Germany

**Keywords:** 16S rRNA gene, high‐throughput sequencing, Illumina, microbial diversity

## Abstract

High‐throughput sequencing of the 16S rRNA gene on the Illumina platform is commonly used to assess microbial diversity in environmental samples. The MiniSeq, Illumina's latest benchtop sequencer, enables more cost‐efficient DNA sequencing relative to larger Illumina sequencing platforms (*e.g.,* MiSeq). Here we used a modified custom primer sequencing approach to test the fidelity of the MiniSeq for high‐throughput sequencing of the V4 hypervariable region of 16S rRNA genes from complex communities in environmental samples. To this end, we designed additional sequencing primers that enabled application of a dual‐index barcoding method on the MiniSeq. A mock community was sequenced alongside the environmental samples in four different sequencing runs as a quality control benchmark. We were able to recapture a realistic richness of the mock community in all sequencing runs, and identify meaningful differences in alpha and beta diversity in the environmental samples. Furthermore, rarefaction analysis indicated diversity in many environmental samples was close to saturation. These results show that the MiniSeq can produce similar quantities of high‐quality V4 reads compared to the MiSeq, yet is a cost‐effective option for any laboratory interested in performing high‐throughput 16S rRNA gene sequencing.

## 1 INTRODUCTION

Continued improvements in DNA sequencing technologies have greatly helped in the democratization of sequencing (Tringe & Hugenholtz, 2008) and high‐throughput sequencing of the 16S rRNA marker gene is widely used to assess diversity and composition of microbial communities (e.g., Bartram, Lynch, Stearns, Moreno‐Hagelsieb, & Neufeld, 2011; Caporaso et al., 2012; Huber et al., 2007; Sogin et al., 2006). However, the start‐up and maintenance costs associated with high‐throughput sequencing still hamper access to these technologies by smaller laboratories.

Illumina's MiniSeq benchtop platform enables cost‐efficient high‐throughput DNA sequencing relative to larger sequencing platforms (*e.g.,* MiSeq). Thus, the goal of this study was to assess the quality of the MiniSeq generated 16S rRNA gene sequence data and to evaluate if this platform is a feasible option for performing 16S rRNA gene high‐throughput sequencing. This would open the possibility for smaller labs to perform their own high‐throughput 16S rRNA gene sequencing, because the MiniSeq is a benchtop sequencer available for ca. 30% of the cost compared to the MiSeq. Furthermore, the reagent kits for the MiniSeq are also ca. 30% the cost of the MiSeq, yet are capable of generating up to 8 million pairs of reads, and the High Output version of this kit produces a volume of sequence data up to 25 million reads (Illumina 2016a).

The dual‐indexed custom primer 16S rRNA gene sequencing protocol for the V4 hypervariable region is widely applied in microbial diversity studies (Kozich, Westcott, Baxter, Highlander, & Schloss, 2013), but was originally developed for sequencing on the MiSeq platform. Thus, our aim was to optimize this dual‐indexed custom primer 16S sequencing protocol for the MiniSeq platform, in order to test the fidelity of the MiniSeq for 16S rRNA gene sequencing. Our modifications to the existing high‐throughput 16S rRNA sequencing protocol (Kozich et al., 2013) use new sequencing primers to adapt this method for the MiniSeq. We performed multiple high‐throughput sequencing runs targeting the V4 hypervariable region of the 16S rRNA gene derived from complex environmental samples, alongside a mock community with a known number of different species. Platform fidelity was assessed by alpha diversity analyses of a mock community of known species composition, which shows that with the proper quality controls the MiniSeq is capable of producing quality 16S rRNA gene sequence data that can be used to rapidly and reliably assess microbial diversity in complex environmental samples.

## 2 MATERIAL AND METHODS

### 2.1 Cultivation and DNA extraction of the 16S mock community

To create a mock community (>3% dissimilarity threshold, see Table S1), pure cultures were isolated from soil, human skin, cell phone swabs, freshwater and saltwater, and grown on agar plates for 3–7 days at room temperature. For genomic DNA extraction, a small amount of each bacterial strain was transferred into a 2 ml sterile lysing Matrix E tube and 800 μl of preheated (60°C) sterile filtered C1 extraction buffer (38 ml saturated NaPO4 [1 mol/L] buffer, 7.5 ml 100% ethanol, 4 ml MoBio's lysis buffer solution C1 [MoBio, Carlsbad, CA], 0.5 ml 10% SDS) was added. The samples were homogenized for 40 s at a speed of 6 m/s using a QuickPrep‐24 5G homogenizer (MP Biomedicals, Santa Ana, CA) and heated for 2 min at 99°C in an Eppendorf ThermoMixer C (Thermo Fisher Scientific, Waltham, MA), followed by two freeze–thaw (−80°C/room temperature) cycles to lyse bacterial cells. After repetition of the homogenizing step, the samples were centrifuged for 10 min at 10,000 g in a Heraeus Pico 21 centrifuge (Thermo Fisher Scientific, Waltham, MA). Microbial DNA was purified using the MoBio PowerClean Pro DNA Clean‐Up Kit (Qiagen, Hilden, Germany) following the manufacturer's instructions using 100 μl of the supernatant. DNA was quantified fluorometrically on the Qubit version 3.0 (Life Technologies, Grand Island, NY) using the Qubit dsDNA high sensitivity assay kit (Life Technologies).

To confirm the number of species in the mock community, the full length 16S rRNA gene of each isolate was amplified and sequenced by Sanger sequencing. Two conserved primers (27f, 1492r) were used to amplify the entire gene during PCR with the following conditions: initial denaturation at 95°C for 3 min; 30 cycles of denaturation at 95°C for 30 s; annealing at 56°C for 30 s; elongation at 72°C for 1 min and a final 5 min extension at 72°C. Individual reactions consisted of 1 μl template DNA, 5 μl 5× Green GoTaq Flexi Buffer (Promega), 3 μl MgCl_2_ (25 mmol/L), 1 μl fw primer (10 μmol/L), 1 μl rv primer (10 μmol/L), 12.9 μl nuclease‐free water, dNTP Mix (10 mmol/L), and 0.1 μl GoTaq Green DNA Polymerase (Promega). The amplicons were subjected to Sanger sequencing using the facilities of the Biocenter of the Ludwig‐Maximilian University (LMU), Martinsried. To confirm dissimilarity thresholds of >3% for all 18 species, we aligned the sequences using BLAST version 2.2.26+ (Altschul, Gish, Miller, Myers, & Lipman, 1990). We pooled the isolates at equimolar concentration and created technical replicates of the mock community to assess the reproducibility of the method.

Environmental samples included salt marsh sediments, freshwater pond sediments, marine sponges, salt water aquaria, and carbonate biofilms (Figure 3). Samples to assess levels of contamination (which were also sequenced, and OTUs removed from the environmental samples) were collected from dust in three different labs in the building where the sequencing and PCR amplifications were performed. Genomic DNA of environmental samples (Run A, *n* = 45; Run B, *n* = 88; Run C, *n* = 84; Run D, *n* = 90) was extracted according to the protocol of Orsi et al. (2017). In brief, samples were transferred to either 50‐ml or 2‐ml Lysing Matrix E tubes containing 1.4 mm ceramic spheres, 0.1 mm silica spheres, and one 4 mm glass sphere (MP Biomedicals, OH) following each incubation. 15 ml (for 50 ml tubes) or 1 ml (for 2 ml tubes) of the extraction buffer (C1 lysing buffer (MoBio, Carlsbad California), 10% SDS, 100% ethanol, and 1 mol/L Na_2_HPO_4_) was added and homogenized for 40 s in a Fast‐Prep 5G homogenizer at a speed of 6 m/s. Then, the supernatant containing the DNA was purified with the DNeasy PowerClean Pro Cleanup Kit (Qiagen, Germany). Extracted DNA was quantified by using the Qubit double‐stranded DNA (dsDNA) high‐sensitivity assay kit and a Qubit 3.0 fluorometer (Invitrogen, Eugene, OR).

### 2.2 16S amplicon library preparation

**Figure 1:**
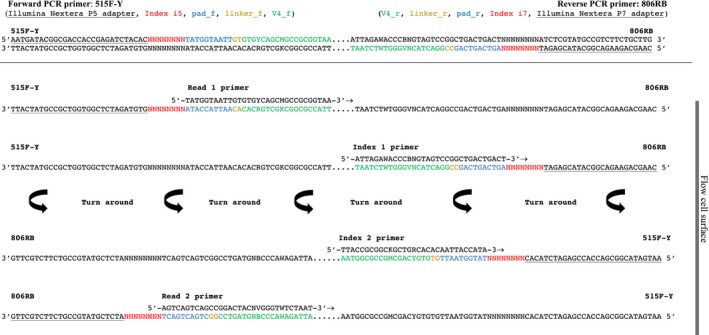
Schematic description of the dual‐index sequencing strategy on the MiniSeq. Reading the figure from top to bottom shows the sequential order of paired‐end sequencing steps (four total). “Turn around” indicates the step of paired‐end turn around on the flow cell surface. The sequencing proceeds in the direction of the flow cell surface, which in this figure is located on the right side (arrows point in direction of sequencing reaction). Sequencing starts by using Read 1 primer to sequence Read 1, followed by Index 1 primer to generate Index 1. The MiniSeq only uses the oligonucleotides on the flow cell for bridging and both the second index and the paired read are sequenced after the clusters are turned around. Hence an Index 2 primer is needed to sequence Index 2. Read 2 is then sequenced by using the Read 2 primer (after Kozich et al., 2013). Sequencing primers for only the forward primer 515F‐Y (Parada et al., 2016) are shown, for sequencing primers needed for the 515F primer (Caporaso et al., 2012) please see Table 1 for all sequencing primers

For Runs A and B, the V4 region of the 16S rRNA gene was amplified with unique barcoded PCR primers 515F (5′ ‐ AATGATACGGCGACCACCGAGATCTACAC NNNNNNNN **TATGGTAATT** GT *GTGCCAGCMGCCGCGGTAA* ‐ 3′) and 806R (5′ ‐ CAAGCAGAAGACGGCATACGAGAT NNNNNNNN **AGTCAGTCAG** CC *GGACTACHVGGGTWTCTAAT* ‐ 3′) (see Table S3 in the supplemental material for barcodes). For Runs C and D, we used modified 515F‐Y/806RB primer constructs (515F‐Y: 5′‐*GTGYCAGCMGCCGCGGTAA*; 806RB: *GGACTACNVGGGTWTCTAAT*), which include the latest changes that increase coverage of Thaumarchaeota (Parada, Needham, & Fuhrman, 2016) and further enable capturing of a greater diversity of the marine SAR11 clade (Apprill, McNally, Parsons, & Weber, 2015) (Table S3). The primer sequences all consist of the appropriate Illumina adapter (P5 or P7; underlined) complementary to the oligonucleotides on the flow cell, an 8‐nt index sequence representing the unique barcode for every sample (N region), a 10‐nt pad sequence (bold), a 2‐nt linker (GT, CC), and the specific primer for the V4 region (italic) (Figure 1). All samples were amplified on the Biometra TProfessional Thermocycler (Biometra, Göttingen, Germany) in a total reaction volume of 24 μl including 2 μl template DNA, 5 μl 5× Green GoTaq Flexi Buffer (Promega), 1 μl forward primer (10 μmol/L), 1 μl reverse primer (10 μmol/L), 1 μl dNTP Mix (10 mmol/L), 3 μl MgCl_2_ (25 mmol/L), 0.2 μl GoTaq Green DNA Polymerase (Promega), and 12.8 μl nuclease‐free water. PCR program was run as follows: initial denaturation at 95°C for 3 min, followed by 30 cycles of denaturation at 95°C for 30 s, annealing at 56°C for 30 s, elongation at 72°C for 1 min and a final elongation step at 72°C for 5 min.

The barcoded DNA amplicons were analyzed on a 1.5% (w/v) agarose gel, and excised and purified for sequencing using the Zymoclean Gel DNA Recovery Kit (Zymo Research, Irvine, CA), adding 15 μl of buffer EB to elute DNA. After gel extraction, DNA concentrations were measured using Qubit and diluted first to 10 nmol/L and then to a final 1 nmol/L in a serial dilution before the samples were pooled (adding 5 μl of every sample).

### 2.3 16S sequencing strategy and primer design

We had to design additional Index 2 sequencing primers (see Table 1) to enable the dual‐index barcoding method on the MiniSeq. Without these additional index sequencing primers on the MiniSeq, it is impossible to demultiplex the samples after the run because the Index 2 sequences will not be sequenced. An additional Index 2 sequencing primer is needed because, as opposed to the MiSeq, the MiniSeq only reads Index 2 after the clusters have been turned around to sequence the pair reads (see Figure 1). Sequencing proceeds in the direction of the flow cell and starts by generating Read 1 (150 bp) using Read 1 sequencing primer, followed by obtaining Index 1 (8 bp) using Index 1 sequencing primer. Clusters are turned around using the oligonucleotides provided on the flow cell. After bridging, Index 2 sequencing primer generates Index 2 (8 bp) and Read 2 sequencing primer finally obtains Read 2 (150 bp).

**Table 1:** Custom sequencing primers used in this study to sequence the 16S rRNA gene (V4 region) amplicons

V4 sequencing primer	Sequence (5′–3′)	Cartridge position	Total volume (μl)	Final concentration (μmol/L)
a)				
Read1.515F	TATGGTAATTGTGTGCCAGCMGCCGCGGTAA	24	16.5	10
Read2.806R	AGTCAGTCAGCCGGACTACHVGGGTWTCTAAT	25	18.3	10
Index1.806R	ATTAGAWACCCBDGTAGTCCGGCTGACTGACT	28	24.6	10
Index2.515F	TTACCGCGGCKGCTGGCACACAATTACCATA	28	25.3	10
b)				
Read1.515F‐Y	TATGGTAATTGTGTGYCAGCMGCCGCGGTAA	24	16.5	10
Read2.806RB	AGTCAGTCAGCCGGACTACNVGGGTWTCTAAT	25	18.3	10
Index1.806RB	ATTAGAWACCCBNGTAGTCCGGCTGACTGACT	28	24.6	10
Index2.515F‐Y	TTACCGCGGCKGCTGRCACACAATTACCATA	28	25.3	10

The primers were diluted and loaded into the specified positions on the Illumina reagent cartridge. We used primers shown in (a) for sequencing Runs A and B, where primers shown in (b) were used for sequencing Runs C and D.

We used the additional Index 2 sequencing primers to perform four paired‐end 16S rRNA sequencing runs on the MiniSeq (Runs A–D). For all runs, we used the MiniSeq Mid Output Reagent Kit (300 cycles) including a reagent cartridge, a single‐use flow cell and hybridization buffer HT1. To prepare our normalized amplicon libraries for sequencing, we followed the MiniSeq Denature and Dilute Libraries Guide (Protocol A) (Illumina 2016d) with some customizations. For run A, we combined 500 μl of the denatured and diluted 16S library (1.8 pM) with 20 μl of denatured and diluted Illumina generated PhiX control library (1.8 pM). The method used to align sequences to PhiX to determine the error profiles is made possible by the software provided by Illumina that is preinstalled on the sequencer. This is calculated automatically by the software after each run, as long as the PhiX has been added to the library loaded into the reagent cartridge.

For Run B, C, and D, we combined 350 μl of the 16S library (1.8 pM) with 150 μl of a denatured and diluted genomic sponge library (*Ephydatia fluviatilis*, 1.8 pM) and additionally added 15 μl of PhiX (1.8 pM). The final 1.8 pM libraries were loaded into the “Load samples” well of the reagent cartridge. For each run, we used four custom sequencing primers Read 1, Index 1, Index 2, and Read 2, which were diluted and loaded into the correct position of the reagent cartridge (see Table 1). The results of the MiniSeq sequencing runs and the 16S rRNA gene sequences from the mock community are publicly available in the ENA Project PRJEB24504.

### 2.4 16S bioinformatics analysis and OTU assignment

Demultiplexing and base calling were both performed using bcl2fastq Conversion Software v2.18 (Illumina, Inc.). All bioinformatics analysis were conducted in USEARCH version 9.2.64 (Edgar, 2010) and QIIME version 1.9.1 (Caporaso et al., 2010). The initial step was to assemble paired‐end reads using the fastq_merge pairs command with default parameters allowing for a maximum of five mismatches in the overlapping region. Stringent quality filtering was carried out using the fastq_filter command. We discarded low‐quality reads by setting the maximum expected error threshold (E_max), which is the sum of the error probability provided by the Q score for each base, to 1. Reads were de‐replicated and singletons discarded. Reads were clustered into OTUs sharing 97% sequence identity using the heuristic clustering algorithm UPARSE (Edgar, 2013), which is implemented in the cluster_otus command. The algorithm performs de novo chimera filtering and OTU clustering simultaneously (Edgar, 2013). The usearch_global command assigned the reads to OTUs and created an OTU table for further downstream analysis. Taxonomy was assigned in QIIME (Caporaso et al., 2010) through BLASTn searches 2.2.26+ (Altschul et al., 1990) with an identity threshold of 90% (hits below 90% identity were not considered) against the SILVA ribosomal RNA gene database (Quast et al., 2013) release for QIIME SILVA123. As a quality control step, we removed all OTUs containing <10 sequences and which had no BLASTn hit. Spurious OTUs were identified in the mock community as those OTUs with a closest BLASTn hit to organisms that were not in the original mock community. The OTU tables were rarefied to the sample containing the lowest number of sequences, with a threshold of >10,000 sequences (all samples having less than 10,000 sequences were removed from analyses prior to the rarefaction step).

### 2.5 16S data analysis

In order to investigate beta diversity structures of our samples, we performed downstream analysis in R version 3.3.0 (R Development Core Team 2011). Nonmetric multivariate (NMDS) analyses of the microbial communities were calculated using a Bray–Curtis distance in the Vegan package (Oksanen et al., 2017). Analysis of Similarity (ANOSIM) was performed using 999 permutations with a Bray–Curtis distance. Rarefaction analyses on environmental samples were performed in QIIME version 1.9.1 using both observed species and chao1 metrics.

## 3 RESULTS AND DISCUSSION

The main modification of our MiniSeq protocol from the dual‐index sequencing method of Kozich et al. (2013) is the use of an additional index sequencing primer. This additional index sequencing primer is necessary because the MiniSeq does not sequence the second index using adapters present on the flow cell surface as the MiSeq does. Rather, the MiniSeq reads Index 2 only after the clusters have been turned around to sequence the paired‐end reads (Figure 1). Thus, in addition to the three sequencing primers described by Kozich et al. (2013), we designed and used new Index 2 sequencing primers, Index2.515F‐Y (5′‐TTACCGCGGCKGCTGRCACACAATTACCATA‐3′) and Index2.515F (5′‐TTACCGCGGCKGCTGGCACACAATTACCATA‐3′) to enable the dual‐index barcoding method on the MiniSeq (see Table 1 for all sequencing primers). We tested this modified approach on four different 16S rRNA sequencing runs including diverse environmental samples as well as a mock community composed of 18 different bacterial species. The mock community was created from pure cultures, whose 16S rRNA genes were determined through Sanger sequencing to be >3% different (Table S1). Environmental samples were collected from salt marsh sediments, freshwater pond sediments, marine sponge, beach sediments, salt water aquaria, and microbial biofilms recovered on carbonate sediments.

### 3.1 Run performances

Run A yielded a total of 1.23 Gbp with cluster density of 76 ± 9 K/mm^2^ and >73% of the clusters passing filter (PF) (Table 2). For Run A, >92% of all bases from both reads were assigned a quality score of Q ≥ 30 with an estimated error rate of 1.37% (Table 2). This first attempt appeared to be under‐clustered considering the low cluster density. According to Illumina's specifications (Illumina 2016b), the recommended cluster density for the mid‐output kit (300 cycles) on the MiniSeq is 170‐220 K/mm^2^. Hence, we optimized cluster density by increasing the genetic diversity of the samples for sequencing runs B, C and D, by spiking in an additional Illumina library of genomic DNA from a marine sponge at a ratio of 1:3 (see Methods).

**Table 2:** Overview of the 16S rRNA sequencing run metrics

		Cycles	Yield	% ≥ Q30	Aligned (%)	Error rate (%)	Cluster PF (%)	Reads (in Mio.)	Reads PF (in Mio.)
Cluster density 76 ± 9 K/mm2	**Run A**								
	Read 1	151	589.25 Mbp	95.18	8.06	1.49	73.28 ± 13.91	5.4	3.9
	Index 1	8	27.50 Mbp	94.86	0.00	0.00	73.28 ± 13.91	5.4	3.9
	Index 2	8	27.48 Mbp	90.50	0.00	0.00	73.28 ± 13.91	5.4	3.9
	Read 2	151	589.04 Mbp	88.96	1.24	1.37	73.28 ± 13.91	5.4	3.9
	Totals	318	1.23 Gbp	92.10	8.02	1.37			
Cluster density 170 ± 3 K/mm^2^	**Run B**								
	Read 1	151	1.58 Gpb	89.12	24.85	0.93	85.65 ± 1.28	12.2	10.5
	Index 1	8	73.73 Mbp	86.14	0.00	0.00	85.65 ± 1.28	12.2	10.5
	Index 2	8	73.74 Mbp	80.91	0.00	0.00	85.65 ± 1.28	12.2	10.5
	Read 2	151	1.58 Gpb	88.58	24.43	0.65	85.65 ± 1.28	12.2	10.5
	Totals	318	3.31 Gpb	88.61	24.64				
Cluster Density 124 ± 1 K/mm^2^	**Run C**								
	Read 1	151	1.28 Gpb	95.48	12.19	0.40	95.52 ± 0.54	8.9	8.5
	Index 1	8	59.56 Mbp	93.75	0.00	0.00	95.52 ± 0.54	8.9	8.5
	Index 2	8	59.57 Mbp	93.39	0.00	0.00	95.52 ± 0.54	8.9	8.5
	Read 2	151	1.28 Gpb	94.21	11.98	0.45	95.52 ± 0.54	8.9	8.5
	Totals	308	2.67 Gpb	94.79	12.09	0.43			
Cluster Density 120 ± 5 k/mm^2^	**Run D**								
	Read 1	151	1.23 Gpb	94.35	8.79	0.93	94.95 ± 1.15	8.6	8.2
	Index 1	8	57.34 Mpb	95.23	0.00	0.00	94.95 ± 1.15	8.6	8.2
	Index 2	8	57.35 Mbp	93.20	0.00	0.00	94.95 ± 1.15	8.6	8.2
	Read 2	151	1.21 Gpb	91.17	8.64	0.65	94.95 ± 1.15	8.6	8.2
	Totals	318	2.56 Gpb	91.87	8.71	0.47			

Runs A and B were performed with primers 515F/806R (Caporaso et al., 2012), and Runs C and D were performed with primers 515F‐Y (Parada et al., 2016) and 806RB (Apprill et al., 2015)

PF: passing filter.

Spiking in the genomic DNA resulted in clusters PF > 80% for Runs B–D, which is expected for optimized cluster density on the platform. For Runs C and D, clustering efficiency of PF > 95% was achieved. For example, sequencing Run B generated 3.31 Gbp with a cluster density of 170 ± 3 K/mm^2^ and >84% of clusters PF (Table 2). Run B had 88% of all bases from both reads assigned a quality score of Q ≥ 30 with an estimated error rate of 0.8%. Run C yielded 2.67 Gbp with a cluster density of 124 ± 1 K/mm^2^ and >95% of the clusters PF (Table 2). For Run C, a Q ≥ 30 was achieved by 94% of all bases, with an estimated error rate of 0.43%. Sequencing Run D generated 2.56 Gbp with a cluster density of 120 ± 51 K/mm^2^ and >94% of the clusters PF (Table 2). In Run D, 93% of bases had a quality score of Q ≥ 30 with an estimated error rate of 0.47%. We note that it is difficult to distinguish sequencing errors from PCR errors, and thus refer to the error rates predicted for the amplicons from the PhiX data as estimated error rates as these do not account for PCR errors.

### 3.2 Terminal G homopolymers

The MiniSeq uses a 2‐channel sequencing by synthesis (SBS) method compared to the 4‐channel SBS technology used on the MiSeq and HiSeq instruments. Clusters appearing in red and green are cytosine (C) and thymine (T) nucleotides, respectively, whereas adenine (A) bases are detected in both channels and appear yellow. Guanine (G) nucleotides are unlabelled clusters and are seen in neither channel hence they appear black (Illumina 2016c).

In our first 16S rRNA sequencing run (Run A) that had relatively poor quality (cluster density 76 ± 9 K/mm^2^, PF < 80%), 7% of forward reads and 8% of reverse reads had long (>10) terminal poly‐G strings (see Figure S1). As G indicates lack of sequencing signal with the Illumina 2‐dye chemistry (*e.g*., black), this may be due to under‐clustering on the flow cell, low diversity in the 16S libraries, or partially amplified V4 PCR fragments carried over during the gel extraction. For this first low‐quality run, we removed all sequences with G homopolymers >10 nucleotides prior to data analysis as the poly‐G homopolymers could apply to all OTUs. Long poly‐G strings were also not detected in the data from the other 16S sequencing runs (Runs B‐D), which had genomic DNA spiked in to increase the nucleotide diversity. Thus, the phenomenon of terminal poly‐G homopolymers appears to be due to the low diversity inherent in 16S sequencing datasets, as this was also not observed in any of our prior genome or transcriptome sequencing libraries on the MiniSeq (data not shown). Thus, we recommend that researchers mix separately indexed genomic libraries together with their 16S rRNA gene libraries when sequencing on the MiniSeq to reduce the number of terminal G homopolymers. Under these conditions, our results show that a cluster density >120 K/mm^2^ and percent of clusters passing filter >90% provide for a high‐quality run. We urge caution when analyzing rare taxa (Sogin et al., 2006) with 16S data generated on the MiniSeq, as the low‐sequencing depth may not be sufficient. Moreover, sequences with terminal poly‐G homopolymers need to be carefully accounted for as they could lead to spurious OTUs. Other modern methods of analysis such as DADA2 (Callahan et al., 2016) could assist with poly‐G containing reads and other erroneous reads in MiniSeq 16S rRNA gene amplicon data.

### 3.3 Mock community analysis

**Figure 2:**
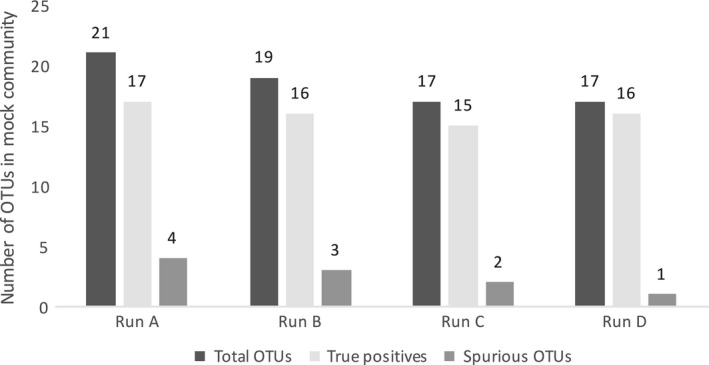
OTU assessment for the mock community composed of 18 defined species. UPARSE generated an accurate estimate of the microbial community in all performed 16S rRNA sequencing runs, given the low number of spurious OTUs

Because the V4 hypervariable region is ca. 250 bp in length the 150 bp pair of reads produced by the MiniSeq overlap 50 bp on average, this may impact diversity estimates (because mismatches in the overlapping contigs are used to assess errors and platform fidelity). We used OTU clustering to see whether the true richness could be recovered in the mock community. For Run A, we had 6 replicates, whereas for Run C and D, 3 replicates were sequenced. After data processing (see Methods), the UPARSE algorithm (Edgar, 2013) recovered 17 of the 18 species in our mock community and 4 spurious OTUs in Run A, 15 species plus 2 spurious OTUs in Run C and 16 species plus 1 spurious one in Run D (Figure 2). The mock community was not sequenced alongside the environmental samples in Run B, but mock community sequences from the other three sequencing runs were clustered together with the environmental samples in Run B to assess the diversity in the generated data set. In this case, the number of species found in the mock community was also close to its true composition (16 out of 18 species, 3 spurious OTUs). Six different bacterial species were found among the spurious OTUs derived from the replicated sequencing runs, of which two were similar (order and family level, respectively) to taxa from the mock community that were not detected. These two spurious OTUs might have been misclassifications due to sequencing errors. The remaining four are presumed to be either contaminants, or derived from sample cross talk. Thus, the UPARSE method could accurately recover the microbial richness from our MiniSeq 16S rRNA gene data. Other studies using UPARSE also showed that the number of OTUs generated with this method is in close concordance with the number of species in mock communities (*e.g*., Edgar, 2013; Flynn, Brown, Chain, MacIsaac, & Cristescu, 2015). While the exact number of OTUs in the mock community was not obtained (Figure 2), mock communities are rarely recovered at the exact richness after 16S high‐throughput sequencing with variability reaching >30% of the richness in the original mock community even under stringent criteria (Edgar, 2013). This is typically attributed to additional undetected contaminants, potential multiple rRNA gene copies harbored by some of the genomes, and single sequencing errors that can occur in low abundance in the sample index barcodes (Edgar, 2016). Furthermore, analysis of a mock community sequenced in parallel with environmental samples is challenging due to sample “cross talk” that can occur partly due to errors in the barcodes themselves (Edgar, 2016). This can occur either during PCR or sequencing, but is difficult to assess if the environmental samples contain similar strains as the mock community. In our case, several of the strains (*Pseudomonas fluorescens*,* Vibrio natriegens*,* Pseudoalteromonas flavipulchra*) in our mock community are closely related to organisms in the environmental samples from marine sediments and corals that were sequenced (Table S1). Thus, it is difficult to speculate on the exact degree of sample cross talk in our sequencing runs.

### 3.4 Analysis of environmental samples

**Figure 3:**
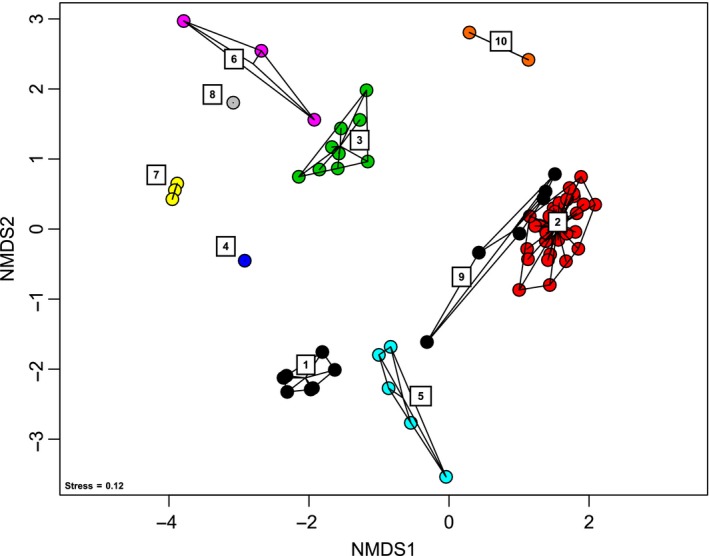
Nonmetric multidimensional scaling analysis showing microbial beta diversity of the 16S data sets. (1) mock community replicates, (2) pond sediments (Niederlibbach, Germany), (3) saltmarsh sediments (Cape Cod, MA), (4) salt water aquaria, (5) marine sponge, (6) sandy beach sediments (Obidos lagoon), (7) microbial mats (Obidos lagoon, Portugal), (8) salt marsh sediments (Pt Judith, RI), (9) pond sediments (Niederlibbach, Germany), and (10) carbonate biofilms (Liguria Springs, Italy)

Our quality control procedures for the MiniSeq 16S rRNA gene data appears to be reasonably prudent, because the richness of our recovered mock community OTUs relative to the starting richness falls within the variability of stringently controlled mock community sequence analyses (Edgar, 2013). To control for contamination, we also sequenced lab dust samples and extraction blanks and removed OTUs shared with the environmental samples. After removal of contaminant OTUs, a significantly different (ANOSIM: *p* = .001, *R*:.9) microbiome for each sample was observed (Figure 3). Given that the richness of the mock community is close to the true value, these beta diversity analyses show that the MiniSeq is a viable platform for high‐throughput 16S rRNA gene sequencing studies of microbiomes.

Furthermore, samples included in the rarefaction analysis reached saturation, indicating the MiniSeq can sample diversity adequately from environmental samples (Figure S2). For each run, there was variability (0.54%–13.91%) in the number of reads (clusters PF) obtained per sample, with Runs C and D having the least variability in sequencing depth between samples (Table 2). Runs C and D were also the highest quality in terms of % clusters passing filter. These data show that even for a sequencing run with 90 samples, after pooling the samples at equimolar concentrations a high‐quality run (>90% of clusters PF) on the MiniSeq can provide upwards of ca. 50,000 reads per sample (Table S2).

For future testing of the fidelity of the MiniSeq for 16S rRNA gene sequencing we encourage other researchers to use mock communities constructed from named isolates with high‐quality genome sequences (e.g., from the DSMZ culture collection: www.dsmz.de), that are not present in the environmental samples. This would reduce potential bias due to sample cross talk with the mock communities, as well as the possibility of multiple 16S rRNA gene copies or paralogs in mock communities constructed de novo that lack complete genome sequences.

Comparing sequencing fidelity across platforms is a feasible way of validating high‐throughput sequencing approaches (Caporaso et al., 2012). However, mock communities can also be used as a way to test the fidelity of high‐throughput sequencing platforms (Benítez‐Páez, Portune, & Sanz, 2016; Caporaso et al., 2011). Thus, while we do not compare our results to those obtained from larger sequencing platforms, for example, a MiSeq (as described by Caporaso et al., 2012), the analyses of the mock community show that the MiniSeq is able to capture a realistic picture of its microbial diversity. For continued testing of the fidelity of the MiniSeq platform for 16S rRNA gene sequencing, future test would benefit from a direct comparison between the same libraries sequenced on both the MiSeq and MiniSeq. For MiniSeq Run D we successfully sequenced 90 samples while yielding ca. 50,000 reads (both forward and reverse) on average per sample. Thus, while the MiniSeq does not provide a sequencing depth equivalent to that of the HiSeq needed for larger scale projects, it represents a new platform for smaller scale sequencing projects (e.g., up to 96 samples, with ca. 50,000 reads per sample) at a reduced per base cost compared to the MiSeq. Our protocol thus increases feasibility for small laboratories to perform their own high‐throughput sequencing of the 16S rRNA marker gene.

## CONFLICT OF INTEREST

The authors declare no conflict of interest.
