# Supplementary figures and images for: A 16S rRNA gene sequencing and analysis protocol for the Illumina MiniSeq platform
Source: Microbiologyopen. 2018 Mar 25;7(6):e00611. doi: 10.1002/mbo3.611 (PMC6291791; doi:10.1002/mbo3.611)

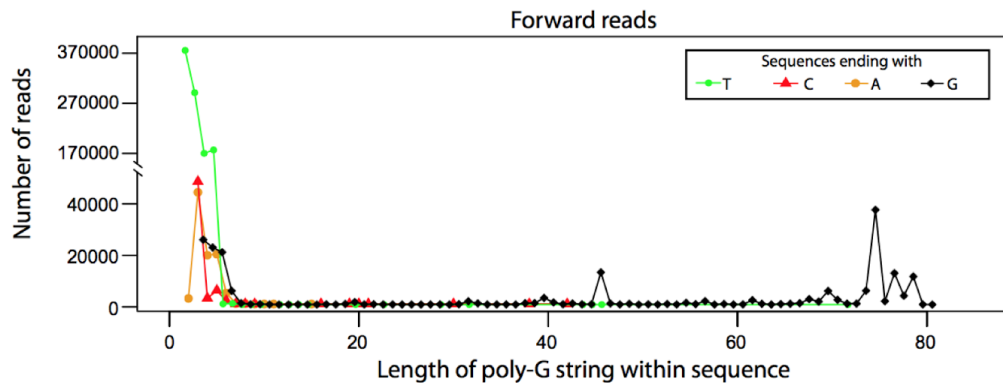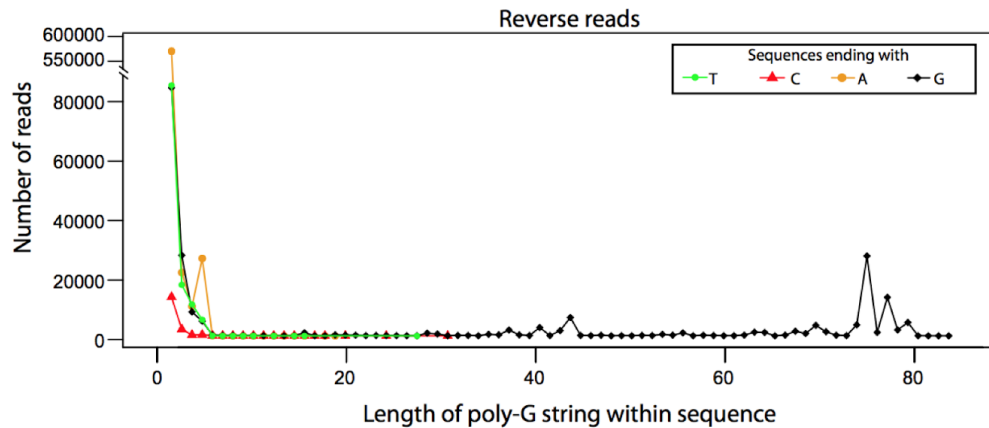

Supplement: Supplementary file 1 [file MBO3-7-e00611-s001.pdf]

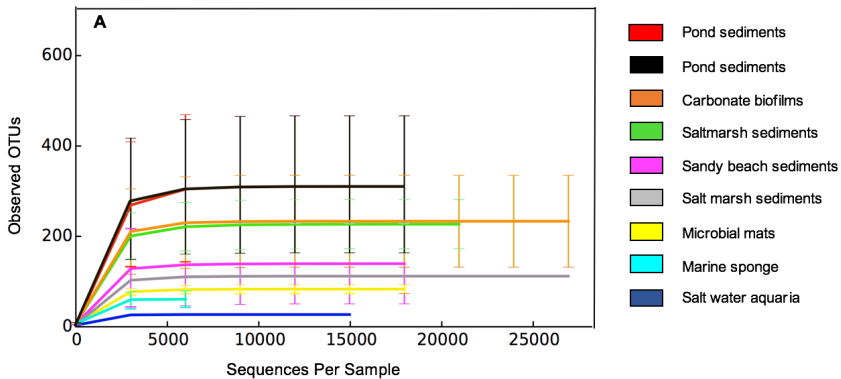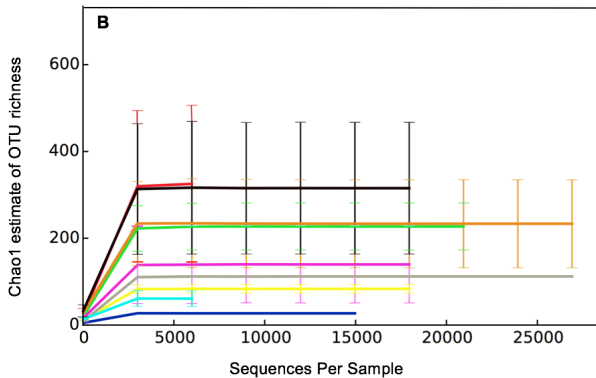

Supplement: Supplementary file 2 [file MBO3-7-e00611-s002.pdf]
