# Supplementary material for: A 16S rRNA gene sequencing and analysis protocol for the Illumina MiniSeq platform
Source: Microbiologyopen. 2018 Mar 25;7(6):e00611. doi: 10.1002/mbo3.611 (PMC6291791; doi:10.1002/mbo3.611)
Supplement: Supplementary file 3 [file MBO3-7-e00611-s003.pdf]

Bacterial Isolate in mock community	Closest hit to GenBank entry	Accession Number	Isolated from	Author	Read numbers		
					**Run A**	**Run C**	**Run D**
Staphylococcus sp.	*Staphylococcus pasteuri*	KX453962.1	no information	Smith et al. 2017	2,077	2,309	6,151
Bacillus sp.	*Bacillus simplex*	KX866679.1	metal contaminated soil	Alaniz-Andrade et al. 2017	4,278	1,425	2,377
Bacillus sp.	*Bacillus sp.*	KX785129.1	no information	Alnaimat 2016	4,673	450	523
Micrococcus sp.	*Micrococcus luteus*	KX866674.1	metal contaminated soil	Alaniz-Andrade et al. 2017	3,204	633	581
Acinetobacter sp.	*Acinetobacter lwoffii*	KX953868.1	sediment	Xiong et al. 2016	6,868	3,221	4,168
Enterobacter sp.	*Enterobacter sp.*	KX364035.1	Quebrada del Zoquete	Cornejo et al. 2016	9,357	5,282	12,360
Aeromonas sp.	*Aeromonas veronii*	KX946876.1	no information	Kang et al. 2016	10,169	5,964	17,779
Carnobacterium sp.	*Carnobacterium maltaromaticum*	KU244600.1	exfoliated sandstone	Zanardini et al. 2016	2,514	1,882	3,834
Exiguobacterium sp.	*Exiguobacterium sibiricum*	KR857420.1	Siberian permafrost	Schuerger et al. 2016	3,187	1,702	4,020
Janthinobacterium sp.	*Janthinobacterium sp.*	LC189077.1	farm soil	Arif et al. 2017	-	-	-
Pseudomonas sp.	*Pseudomonas fluorescens*	KT695823.1	Wyoming Soil	Tao 2015	2,735	3,548	4,084
Photobacterium sp.	*Photobacterium rosenbergii*	KP843685.1	coral mucus	Embarcadero-Jimenez et al. 2015	5,938	2,082	4,916
Pseudoalteromonas sp.	*Pseudoalteromonas flavipulchra*	LC189370.1	coral	Wijayanti et al. 2016	697	126	267
Vibrio sp.	*Vibrio natriegens*	KT986142.1	ocean water	Jiang et al. 2015	1,019	500	1,059
Rhodococcus sp.	*Rhodococcus sp.*	KY970076.1	soil	Chitranshi et al. 2017	558	171	110
Sphingobium sp.	*Sphingobium sp.*	KY927392.1	soil	Cai 2017	38	-	118
Arthrobacter sp.	*Arthrobacter sp.*	KY970072.1	soil	Chitranshi et al. 2017	48	-	-
Mycobacterium sp.	*Mycobacterium sp.*	KX509812.1	activated sludge	Zhang and Yuan 2016	353	151	85
