# Supplementary material for: A 16S rRNA gene sequencing and analysis protocol for the Illumina MiniSeq platform
Source: Microbiologyopen. 2018 Mar 25;7(6):e00611. doi: 10.1002/mbo3.611 (PMC6291791; doi:10.1002/mbo3.611)
Supplement: Supplementary file 4 [file MBO3-7-e00611-s004.pdf]

Run A (n= 45)		Run B (n=88)		Run C (n=84)		Run D (n=90)	
Sample ID	Number of reads	Sample ID	Number of reads	Sample ID	Number of reads	Sample ID	Number of reads
RunA_S1	62624	RunB_S1	903	RunC_S1	52705	RunD_S1	77382
RunA_S2	74665	RunB_S2	10448	RunC_S2	40189	RunD_S2	44442
RunA_S3	96853	RunB_S3	47252	RunC_S3	44317	RunD_S3	42990
RunA_S4	26569	RunB_S4	13438	RunC_S4	40281	RunD_S4	70156
RunA_S5	25015	RunB_S5	32902	RunC_S5	29564	RunD_S5	40760
RunA_S6	34513	RunB_S6	42514	RunC_S6	56339	RunD_S6	60442
RunA_S7	28703	RunB_S7	38340	RunC_S7	67306	RunD_S7	65848
RunA_S8	29579	RunB_S8	38333	RunC_S8	45029	RunD_S8	37629
RunA_S9	37268	RunB_S9	24188	RunC_S9	70277	RunD_S9	23115
RunA_S10	43048	RunB_S10	32288	RunC_S10	60842	RunD_S10	19623
RunA_S11	45926	RunB_S11	82384	RunC_S11	68600	RunD_S11	32095
RunA_S12	15992	RunB_S12	42174	RunC_S12	53269	RunD_S12	23154
RunA_S13	14791	RunB_S13	42422	RunC_S13	48449	RunD_S13	20503
RunA_S14	11257	RunB_S14	88214	RunC_S14	57298	RunD_S14	49803
RunA_S15	15971	RunB_S15	28259	RunC_S15	53339	RunD_S15	36020
RunA_S16	12933	RunB_S16	41928	RunC_S16	54889	RunD_S16	33545
RunA_S17	51114	RunB_S17	37282	RunC_S17	64781	RunD_S17	54064
RunA_S18	108533	RunB_S18	38427	RunC_S18	61323	RunD_S18	46676
RunA_S19	83093	RunB_S19	40759	RunC_S19	60001	RunD_S19	39764
RunA_S20	62856	RunB_S20	28059	RunC_S20	72945	RunD_S20	49826
RunA_S21	54155	RunB_S21	38081	RunC_S21	82476	RunD_S21	17649
RunA_S22	58227	RunB_S22	100269	RunC_S22	48229	RunD_S22	39956
RunA_S23	57331	RunB_S23	19246	RunC_S23	70187	RunD_S23	46447
RunA_S24	69482	RunB_S24	24480	RunC_S24	23731	RunD_S24	45222
RunA_S25	12306	RunB_S25	29927	RunC_S25	41029	RunD_S25	39645
RunA_S26	12154	RunB_S26	107917	RunC_S26	49626	RunD_S26	37400
RunA_S27	17795	RunB_S27	64135	RunC_S27	36169	RunD_S27	46212
RunA_S28	34774	RunB_S28	76448	RunC_S28	37065	RunD_S28	40735
RunA_S29	19584	RunB_S29	98246	RunC_S29	35770	RunD_S29	45999
RunA_S30	30815	RunB_S30	87179	RunC_S30	55011	RunD_S30	35807
RunA_S31	38841	RunB_S31	72001	RunC_S31	41446	RunD_S31	44480
RunA_S32	33137	RunB_S32	45491	RunC_S32	44382	RunD_S32	39174
RunA_S33	34370	RunB_S33	28360	RunC_S33	30983	RunD_S33	54194
RunA_S34	39898	RunB_S34	37259	RunC_S34	63539	RunD_S34	25674
RunA_S35	38655	RunB_S35	78820	RunC_S35	75552	RunD_S35	145
RunA_S36	31711	RunB_S36	8604	RunC_S36	73336	RunD_S36	26726
RunA_S37	41549	RunB_S37	22094	RunC_S37	43057	RunD_S37	45071
RunA_S38	143835	RunB_S38	38635	RunC_S38	40319	RunD_S38	46334
RunA_S39	99682	RunB_S39	58191	RunC_S39	32651	RunD_S39	38855
RunA_S40	105959	RunB_S40	22933	RunC_S40	37460	RunD_S40	26081
RunA_S41	60506	RunB_S41	93466	RunC_S41	55309	RunD_S41	49347
RunA_S42	21486	RunB_S42	13467	RunC_S42	40375	RunD_S42	19980
RunA_S43	41261	RunB_S43	34326	RunC_S43	40542	RunD_S43	35536
RunA_S44	42373	RunB_S44	36549	RunC_S44	38441	RunD_S44	31199
RunA_S45	30657	RunB_S45	27186	RunC_S45	42819	RunD_S45	27284
		RunB_S46	35796	RunC_S46	51529	RunD_S46	20477
		RunB_S47	28818	RunC_S47	92500	RunD_S47	34799
		RunB_S48	40656	RunC_S48	41511	RunD_S48	43784
		RunB_S49	12119	RunC_S49	33872	RunD_S49	35595
		RunB_S50	658	RunC_S50	41195	RunD_S50	36919
		RunB_S51	19830	RunC_S51	48704	RunD_S51	27295
		RunB_S52	24256	RunC_S52	37620	RunD_S52	48307
		RunB_S53	28472	RunC_S53	276535	RunD_S53	27292
		RunB_S54	27282	RunC_S54	47288	RunD_S54	31990
		RunB_S55	24263	RunC_S55	29418	RunD_S55	54744

RunB_S56	20963	RunC_S56	41442	RunD_S56	37450
RunB_S57	27840	RunC_S57	37227	RunD_S57	48802
RunB_S58	11444	RunC_S58	35556	RunD_S58	29435
RunB_S59	43028	RunC_S59	37488	RunD_S59	40799
RunB_S60	13289	RunC_S60	39816	RunD_S60	36325
RunB_S61	26108	RunC_S61	53840	RunD_S61	135
RunB_S62	19343	RunC_S62	31499	RunD_S62	33337
RunB_S63	13134	RunC_S63	34086	RunD_S63	44584
RunB_S64	17771	RunC_S64	43171	RunD_S64	41374
RunB_S65	19605	RunC_S65	47683	RunD_S65	36309
RunB_S66	19720	RunC_S66	33814	RunD_S66	36774
RunB_S67	19893	RunC_S67	75834	RunD_S67	28799
RunB_S68	15535	RunC_S68	30846	RunD_S68	43452
RunB_S69	39560	RunC_S69	48541	RunD_S69	35012
RunB_S70	22353	RunC_S70	42245	RunD_S70	49785
RunB_S71	70	RunC_S71	49643	RunD_S71	37114
RunB_S72	25699	RunC_S72	42148	RunD_S72	32699
RunB_S73	30478	RunC_S73	46945	RunD_S73	32342
RunB_S74	9770	RunC_S74	29452	RunD_S74	31489
RunB_S75	15375	RunC_S75	21602	RunD_S75	25181
RunB_S76	3088	RunC_S76	45180	RunD_S76	36205
RunB_S77	23934	RunC_S77	42138	RunD_S77	22767
RunB_S78	23672	RunC_S78	40285	RunD_S78	35514
RunB_S79	19002	RunC_S79	58752	RunD_S79	22931
RunB_S80	13135	RunC_S80	42129	RunD_S80	35027
RunB_S81	23816	RunC_S81	40323	RunD_S81	37786
RunB_S82	14529	RunC_S82	46586	RunD_S82	32680
RunB_S83	23296	RunC_S83	50941	RunD_S83	41451
RunB_S84	14597	RunC_S84	49194	RunD_S84	33092
RunB_S85	33670			RunD_S85	39299
RunB_S86	35730			RunD_S86	31063
RunB_S87	28797			RunD_S87	388
RunB_S88	22130			RunD_S88	34466
				RunD_S89	36316
				RunD_S90	29444
