# Supplementary material for: A 16S rRNA gene sequencing and analysis protocol for the Illumina MiniSeq platform
Source: Microbiologyopen. 2018 Mar 25;7(6):e00611. doi: 10.1002/mbo3.611 (PMC6291791; doi:10.1002/mbo3.611)
Supplement: Supplementary file 5 [file MBO3-7-e00611-s005.pdf]

PCR Primer	Barcode	Sequence (5’ to 3')
**515F-Y.A501**	ATCGTACG	AATGATACGGCGACCACCGAGATCTACACATCGTACGTATGGTAATTGTGTGYCAGCMGCCGCGGTAA
**515F-Y.A502**	ACTATCTG	AATGATACGGCGACCACCGAGATCTACACACTATCTGTATGGTAATTGTGTGYCAGCMGCCGCGGTAA
**515F-Y.A503**	TAGCGAGT	AATGATACGGCGACCACCGAGATCTACACTAGCGAGTTATGGTAATTGTGTGYCAGCMGCCGCGGTAA
**515F-Y.A504**	CTGCGTGT	AATGATACGGCGACCACCGAGATCTACACCTGCGTGTTATGGTAATTGTGTGYCAGCMGCCGCGGTAA
**515F-Y.A505**	TCATCGAG	AATGATACGGCGACCACCGAGATCTACACTCATCGAGTATGGTAATTGTGTGYCAGCMGCCGCGGTAA
**515F-Y.A506**	CGTGAGTG	AATGATACGGCGACCACCGAGATCTACACCGTGAGTGTATGGTAATTGTGTGYCAGCMGCCGCGGTAA
**515F-Y.A507**	GGATATCT	AATGATACGGCGACCACCGAGATCTACACGGATATCTTATGGTAATTGTGTGYCAGCMGCCGCGGTAA
**515F-Y.A508**	GACACCGT	AATGATACGGCGACCACCGAGATCTACACGACACCGTTATGGTAATTGTGTGYCAGCMGCCGCGGTAA
**806RB.A701**	AACTCTCG	CAAGCAGAAGACGGCATACGAGATAACTCTCGAGTCAGTCAGCCGGACTACNVGGGTWTCTAAT
**806RB.A702**	ACTATGTC	CAAGCAGAAGACGGCATACGAGATACTATGTCAGTCAGTCAGCCGGACTACNVGGGTWTCTAAT
**806RB.A703**	AGTAGCGT	CAAGCAGAAGACGGCATACGAGATAGTAGCGTAGTCAGTCAGCCGGACTACNVGGGTWTCTAAT
**806RB.A704**	CAGTGAGT	CAAGCAGAAGACGGCATACGAGATCAGTGAGTAGTCAGTCAGCCGGACTACNVGGGTWTCTAAT
**806RB.A705**	CGTACTCA	CAAGCAGAAGACGGCATACGAGATCGTACTCAAGTCAGTCAGCCGGACTACNVGGGTWTCTAAT
**806RB.A706**	CTACGCAG	CAAGCAGAAGACGGCATACGAGATCTACGCAGAGTCAGTCAGCCGGACTACNVGGGTWTCTAAT
**806RB.A707**	GGAGACTA	CAAGCAGAAGACGGCATACGAGATGGAGACTAAGTCAGTCAGCCGGACTACNVGGGTWTCTAAT
**806RB.A708**	GTCGCTCG	CAAGCAGAAGACGGCATACGAGATGTCGCTCGAGTCAGTCAGCCGGACTACNVGGGTWTCTAAT
**806RB.A709**	GTCGTAGT	CAAGCAGAAGACGGCATACGAGATGTCGTAGTAGTCAGTCAGCCGGACTACNVGGGTWTCTAAT
**806RB.A710**	TAGCAGAC	CAAGCAGAAGACGGCATACGAGATTAGCAGACAGTCAGTCAGCCGGACTACNVGGGTWTCTAAT
**806RB.A711**	TCATAGAC	CAAGCAGAAGACGGCATACGAGATTCATAGACAGTCAGTCAGCCGGACTACNVGGGTWTCTAAT
**806RB.A712**	TCGCTATA	CAAGCAGAAGACGGCATACGAGATTCGCTATAAGTCAGTCAGCCGGACTACNVGGGTWTCTAAT
